# The rewarding compensatory mechanism of music enhances the sense of fairness

**DOI:** 10.3389/fnbeh.2022.890739

**Published:** 2022-08-01

**Authors:** Zhengxian Liu, Lan Yang, Siyu Long, Junce Wang, Yajing Si, Lihui Huang, Binxin Huang, Rui Ding, Jing Lu, Dezhong Yao

**Affiliations:** ^1^The Clinical Hospital of Chengdu Brain Science Institute, University of Electronic Science and Technology of China, Chengdu, China; ^2^Center for Information in Medicine, School of Life Sciences and Technology, University of Electronic Science and Technology of China, Chengdu, China; ^3^Faculty of Education, Southwest University, Chongqing, China; ^4^College of International Education, Sichuan International Studies University, Chongqing, China; ^5^School of Psychology, Xinxiang Medical University, Xinxiang, China; ^6^Institute of Brain and Psychological Sciences, Sichuan Normal University, Chengdu, China; ^7^Research Unit of NeuroInformation, Chinese Academy of Medical Sciences, Chengdu, China; ^8^School of Electrical Engineering, Zhengzhou University, Zhengzhou, China

**Keywords:** music, moral judgment, ultimatum game, fair, brain network

## Abstract

Whether music can influence moral judgment is controversial in the aesthetics and philosophy of music. Aesthetic Autonomy pointed out that music had a morally educational function because of its lyrics or a particular context. The key to resolving the divergence is to select absolute music without lyrics or specific context as the eliciting material. In this study, 84 participants were recruited and randomly divided into three groups to complete the Ultimatum Game (UG) after listening to different stimuli: absolute music, white noise, and no sound. Behavioral results indicated that the participants’ acceptance of unfair offers was significantly lower in the music group. Also, participants in the music group have a shorter reaction time for rejecting an unfair offer than other unfair conditions. However, ERP comparison showed no significant difference in medial frontal negativity (MFN) amplitude, which reflects fairness levels, between the music group and the no sound group for either accepting or rejecting the moderately unfair offer. Brain network analyses revealed that participants in the music group showed stronger activation of rewarding circuits, including the ventral striatum, during the decision-making process of rejecting unfair offers, before the decision especially, compared to the no sound group. These results suggest that absolute music can influence fair decision-making. The reward activated by music compensates participants vicariously for the reward they receive for choosing self-interest in an unfair offer, participants no longer have to choose between self-interest and fairness norms, so the participants reject the unfair offer due to the negative emotions induced by the unfair offer.

## Introduction

Music is a social product (Davidson, 2004). Despite the vast cultural differences, both China and the West have valued the social function of music in education and governance as an important tool for maintaining social order (Plato, 1969; Cook, 1995; Aristotle, 1998; Brown and Volgsten, 2005; Wilson, 2018; Sarasso et al., 2022). However, since the Enlightenment, influenced by the aesthetics of Kant and Hegel, some researchers have suggested that the field of art is essentially independent of the area of morality, questioning the notion of art as a tool for moral education. Based on Kant’s division between autonomy and heteronomy, Gatz distinguished two opposing schools of musical aesthetics. The Aesthetic Heteronomy believed that the meaning and value of music existed outside the work itself, and that art was subordinate to social and political needs. A typical representative of Aesthetic Heteronomy is the socialist-realist view of music. According to Tolstoy, the function of art was that artists convey specific emotions to the audience most directly and powerfully. In contrast, Aesthetic Autonomy, which views musical works as autonomous objects, argued that the laws of music could only be found in the music itself (Gatz, 1929). Kivy suggests that absolute music (music with no lyrics and no specific background) simply does not possess the materials necessary to arouse emotions in listeners in any artistically relevant way (Kivy, 2009).

With both sides of the debate making value judgments based on different positions of musical independence, we need to examine whether music, the absolute music in particular, can objectively influence moral judgments on a factual level. Because Kivy pointed out that music had a moral educational function because of its lyrics or a specific context. So, if we confirm absolute music can influence moral judgments based on experiments, we could provide further insights on the debate over whether music can influence moral judgments.

With the rise of the emotional revolution in psychology, researchers have discussed the impact of music on social decision-making, and the findings all suggested that music can influence moral decision-making, such as antisocial behavior (Anderson et al., 2003), pro-social behavior (Greitemeyer, 2009, 2011; Seidel and Prinz, 2013; Ruth, 2018, 2019), gender bias (Fischer and Greitemeyer, 2006; Greitemeyer et al., 2015), ethnic stereotyping (LaMarre et al., 2012), lying (Ziv et al., 2012; Seidel and Prinz, 2013), obedience (Ziv, 2016), and moral judgments (Skulmowski et al., 2014; Steffens, 2020). Although these topics are more or less related to morality, however, given the diversity and cross-cultural differences of moral topics, we need to select typical moral topics with cross-culturally consistency. Fairness is one of the six moral foundations of Haidt’s Moral Foundations Theory and has been identified to have broad cross-cultural consistency (Graham et al., 2011). The Ultimatum Game (UG) is also seen as an ideal task paradigm for testing individual responses to the fairness of resource allocation (Güth et al., 1982).

In the UG, one player (the proposer) receives an amount of money that she or he has to divide between herself or himself and another player (the responder). If the other player (responder) accepts the division scheme, the split is made according to the proposer’s offer. However, if the responder rejects, both parties receive nothing. Studies using UG have shown that participants prone to reject unfair offers, especially those below 20% of the total (Sanfey et al., 2003). This result challenges the economic rational man assumption. In the UG, responders need to choose between self-interest and fairness norms (Sanfey et al., 2003; Tabibnia et al., 2008; Speitel et al., 2019).

Given Kivy’s explicit suggestion that absolute music is the key to resolving the divergence, the previous studies have not selected absolute music as the eliciting material consciously, and thus are unable to confirm whether it is the music itself, such as timbre, pitch, rhythm, or the lyrics (Anderson et al., 2003; Greitemeyer, 2009, 2011; Greitemeyer et al., 2015; Ruth, 2019), musical context (LaMarre et al., 2012; Lang et al., 2016), etc., influenced the outcome of moral judgments.

Researches on music-induced emotion suggested that absolute music can give us pleasure (Azcarate, 2017; Di Bona, 2018), even atonal music can lead to pleasure (Mencke et al., 2019). Brattico et al. (2011) discovered that happy music without lyrics induced stronger positive emotions than happy music with lyrics. Happy music without lyrics activated structures of the limbic system and the right pars opercularis of the inferior frontal gyrus, whereas auditory regions alone responded to happy music with lyrics. In addition, Greene et al. (2001) and Koenigs et al. (2007) identified the critical role of emotions in moral judgment by fMRI. Researchers have explored the influences of three types of emotions (i.e., the integral emotion experienced at the time of decision making, the incidental emotion aroused by a task-unrelated dispositional or situational source, and the interaction of emotion and cognition) on fairness-related decision making (Zheng et al., 2017). So, we hypothesized that absolute music could influence moral decision-making, and the happy emotions induced by absolute music (incidental emotions) reduced the negative emotions (integral emotions) aroused by the unfair offer, thus leading to a greater willingness to accept the unfair offer after listening to the music.

In this study, absolute music was selected from the Chinese Affective Music System (CAMS) as the eliciting material (Li et al., 2012), and Participants completed UG after listening to the music. EEG techniques were used to explore the neural activity underlying the problem, so as to reveal the mechanism of absolute music that influences fair decision-making.

## Materials and methods

### Participants

Eighty-four healthy right-handed participants were recruited from the University of Electronic Science and Technology of China. None of them were musicians nor previously had the long-lasting experience of music instrument training. The experiment was conducted in accordance with the Declaration of Helsinki and was approved by the Ethics Committee of the University of Electronic Science and Technology of China. The participants were randomly assigned to three groups. The music group and the white noise group participate in the UG after listening to either music or white noise. 28 participants (15 males, 13 females, age 22.75 ± 1.58) listened to the music, 28 participants (15 males, 13 females, age 22.82 ± 2.48) as the active control group listened to the white noise, 28 participants (12 males, 16 females, age 22.64 ± 2.75) as a passive control group performed the UG directly in the no sound group. An *a priori* sample size estimation was conducted using G*Power v.3.1.9.7 (Faul et al., 2009). According to the analysis [*d* = 0.25, α = 0.05, β = 0.95, analysis of variance (ANOVA): repeated measures, within-between interaction], a total sample size of 45 participants was required to detect a reliable effect. All participants provided informed consent and were monetarily compensated for their participation.

### Stimuli

According to the study of Juslin et al. (2011), 85% of listeners listen to music for relaxation. Ten emotions such as happy, sad, and calm are the most common musical emotions, with happy emotions being much more frequent than other musical emotions. Listeners value music primarily for its ability to arouse pleasurable emotions (Juslin et al., 2011). Thus, we selected one piece of music (No. HAPPY01) from CAMS as the music material to induce positive emotions. The white noise in the control group was the sound of rain.

### Experimental protocol

The participants were tested individually, and the whole process lasted about 1 h. During the experiment, participants first completed the online information collection on music preferences and music training experiences. Afterward, participants received general instructions about the UG. The EEG session consisted of a 5-min resting EEG data collection and a 15-min UG task state data collection. Given that studies have indicated that if participants perform a cognitive task while listening to music, music listening will consume resources that could be otherwise dedicated to the task (Thompson et al., 2012), so the UG was performed after listening to 1 min of music or white noise. To measure changes in emotion before and after listening to music or white noise, participants were asked to complete a separate mood rating on a 5-point Likert scale before (mood rating 1) and after (mood rating 2) listening to music or white noise. Participants were presented with the statements: “Currently, I am in a good mood,” “As I answer these questions, I feel cheerful,” “For some reason, I am not very comfortable right now,” and “At this moment, I feel sad” (Peterson and Sauber, 1983). The values for the third and fourth questions were reverse coded, and the sum of the four question scores constituted an emotional score, with higher scores indicating happier emotions. Reliability analyses indicated that each mood metric was internally consistent (mood 1: α = 0.693; mood 2: α = 0.740).

**FIGURE 1:**
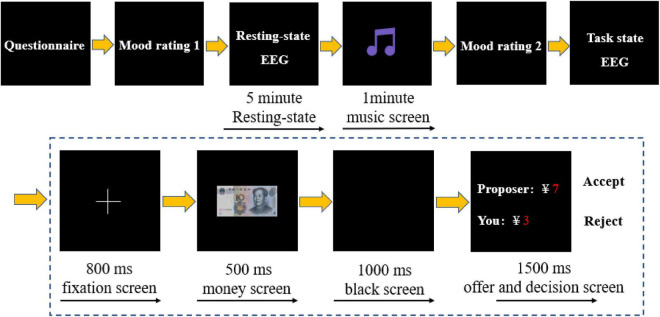
The experimental protocol.

After the participant completed the online questionnaire, the next same-gender participant arrived in the lab to complete the same instructions. The companion sited at a separate table away from the participant, visually blocking their communication, but the participant could hear the interaction between the companion and the experimenter. The companion was then taken to the next lab for preparing (washing the scalp, etc.). The purpose of this was to make all participants think that they were completing the UG with a real partner. However, all offers were predefined. Participants were always assigned to the role of the responder, even though they were ostensibly told that the assignment was random, to examine participants’ responses to unfair offers. The sum of splits was ¥10. The participants received 5 categories pre-determined offers: ¥5: ¥5; ¥6: ¥4; ¥7: ¥3; ¥8: ¥2; ¥9: ¥1, with each category appearing 40 times randomly, for a total of 200 trials throughout the task. Participants decided to accept or reject the offer by pressing either the F or J key with their left or right index finger. E-Prime 3.0 software (Psychology Software Tools, Pittsburgh, PA, United States) was used for stimulus presentation and behavioral data collection. The experimental protocol is shown in Figure 1.

### EEG date recording

The EEG recording was performed with 64 Ag/AgCl electrodes (ANT Neuro, Germany), which were situated according to the extended 10–20 system, and the data were recorded at the sample rate of 1,000 Hz. The bandpass filter was set at 0.3–100 Hz, and CPz served as the reference. Electrooculograms (EOGs) were recorded from an additional channel on the left eye to monitor eye movements. During the entire experimental task, the impedance of the electrodes was maintained below 10 kΩ.

### EEG pre-processing

Firstly, all data were re-referenced to zero reference by the reference electrode standardization technique^1^ (Yao, 2001), followed by the 1–20 Hz bandpass filter. Secondly, we used independent component analysis to remove eye movement interference from EEG data. Thirdly, the data were segmented from −200 to 800 ms (0ms denotes offer onset). Then we excluded the bad trials whose extreme values were over ±80 μV. Finally, we corrected the baseline (−200 to 0 ms) of each trial.

### Medial frontal negativity

Medial frontal negativity (MFN), also known as feedback-related negativity (FRN), is an EEG component that reflects levels of fairness and is widely used in decision-making research. MFN peaks at about 200–350 ms after the onset of the outcome (peaks at about 200–350 ms post outcome onset), reflecting whether the outcome conforms to social norms and is sensitive to the extent to which the outcome deviates from expectations. During UG, unfairness conditions induce larger MFN magnitudes compared to fairness conditions (Gehring and Willoughby, 2002; Hewig et al., 2011; Wu et al., 2011; Massi and Luhmann, 2015; Hu and Mai, 2021).

### Brain network analyses

To explain the role and mechanisms of music in fair decision-making, we chose the Brainstorm^2^ (Tadel et al., 2011) toolbox in Matlab (Mathworks Inc., Natick, MA, United States) for the computation of the brain source-space functional connectivity. The standard International Consortium for Brain Mapping (ICBM) 152 anatomy included in Brainstorm was used to construct the head model for source imaging analysis across all the subjects. The selection of region of interest (ROI) is based on a review of emotions influencing fair decision-making (Zheng et al., 2017). During distributed source imaging analysis, current dipoles were located at the vertices of the cortical surface models and constrained to orientation orthogonal to the cortical surface. A boundary element method (BEM) model was generated using OpenMEEG’s symmetric BEM technique (Gramfort et al., 2010). Then all subject’s electrodes were registered to the head model and used together with the BEM model to compute a lead-field matrix for each subject.

The noise covariance matrix was computed based on the average trial’s time series of all the subjects in each group under each particular task condition prior to source imaging. Standardized low-resolution brain electromagnetic tomography (sLORETA) was used in the source imaging (Pascual-Marqui, 2002). The brain network diagram of source localization was drawn using the BRAPH toolkit^3^ (Mijalkov et al., 2017).

### Phase loking value

The phase locking value (PLV) can measure the phase synchronization between two narrow-band signals. The PLV between signals *s1(t)* and *s2(t)* was formulized as follows.

First, we used the Hilbert transform:


(1) $$\begin{array}{c} Z_{i}\,\left(t\right)=s_{i}\,\left(t\right)+j\,H\,T\,\left(s_{i}\,\left(t\right)\right)\,\#\,\left(7\right) \end{array}$$

Where *HT*(*s*_*i*_(*t*)) is the Hilbert transform of *s*_*i*_(*t*) defined as


(2) $$\begin{array}{c} H\,T\,\left(S_{i}\,\left(t\right)\right)=\frac{1}{\pi }\,P.V.\int_{-\infty }^{\infty }\frac{s_{i}\,\left(t\right)}{t-\tau }\,d\tau \,\#\,\left(8\right) \end{array}$$

And P.V. denotes Cauchy principal value. Once the analytic signals are defined, the constant phase between *Z*_1_(*t*) and *Z*_2_(*t*) can be computed as


(3) $$\begin{array}{c} \Delta \,\phi \,\left(t\right)=\arg\left(\frac{z_{1}\,\left(t\right)\,z_{2}^{*}\,\left(t\right)}{\left|z_{1}\,\left(t\right)\right|\,\left|z_{2}\,\left(t\right)\right|}\right)\,\#\,\left(9\right) \end{array}$$

The instantaneous PLV is then defined as (Lachaux et al., 1999; Celka, 2007)


(4) $$\begin{array}{c} P\,L\,V\,\left(t\right)=\left|E\,\left[e^{j\,\Delta \,\phi \,\left(t\right)}\right]\right|\,\#\,\left(10\right) \end{array}$$

*E[.]* represents the expected value. We selected nine regions of interest and calculated the difference map based on PLV in different groups.

## Results

### Behavioral results

#### Manipulation check: mood induction

Paired *t*-tests of the participants’ two mood ratings before and after listening to the music indicated that happy music effectively induced happy emotion (*t* = −2.542, *p* = 0.017).

To confirm that music is actively changing participants’ mood, analyses of variance (ANOVA) were conducted on emotional scores with time (pre- vs. post-induction) as the within-subject factor and group as the between-subject factor. Table 1 shows that, before listening to music/white noise, there was no significant difference between the emotional scores of the music and white noise groups (*p* = 0.877); however, after listening to music/white noise, there was a significant difference between the emotional scores of the music and white noise groups (*p* < 0.001).

**TABLE 1:** Difference in emotional scores between the music and white noise groups.

	Music	White noise	*F*	*P*
Pre- induction	15.43 ± 2.5	15.54 ± 2.66	0.024	0.877
Post-induction	17 ± 2.16	11.86 ± 3.5	43.713***	0.000

***P < 0.001.

#### Acceptance rates

Table 2 illustrates the ARs of offers in the UG. Mann–Whitney *U* test showed that participants in the music group had significantly lower ARs in the moderately unfair offer of 4 (*z* = −2.164, *n* = 56, *p*_[FDR]_ = 0.032) and 3 (*z* = −2.149, *n* = 56, *p*_[FDR]_ = 0.032) than participants in the no sound group. However, there were no significant differences in ARs between the music and white noise groups, or between the white noise and no sound groups for any offers (all *P*s > 0.05). Thus, in the subsequent analyses, we only focused on the differences between the music and no sound groups in the moderately unfair offer of 4 and 3 (The comparisons of music group vs. white noise group and the white noise group vs. no sound group are supplied in the Supplementary material).

**TABLE 2:** Difference in acceptance rate among different experimental groups.

	Music	White noise	No sound
¥5: ¥5 Acceptance rate	0.9446	0.9643	0.9759
¥6: ¥4 Acceptance rate	0.4438	0.6196	0.6688
¥7: ¥3 Acceptance rate	0.2036	0.2705	0.3848
¥8: ¥2 Acceptance rate	0.0696	0.0491	0.1473
¥9: ¥1 Acceptance rate	0.0205	0.0268	0.0330

#### Reaction time

**FIGURE 2:**
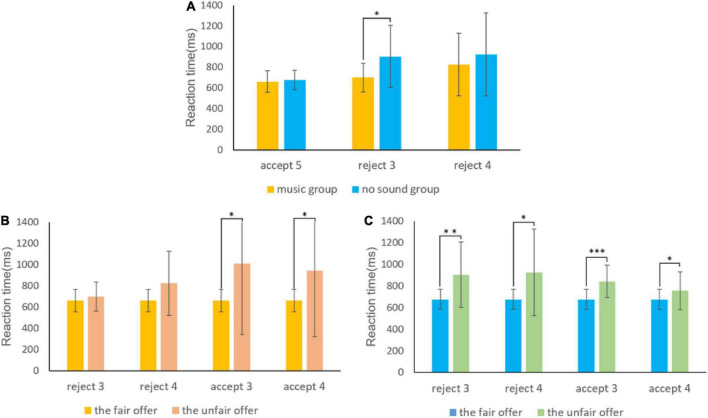
Reaction time in different conditions. Panel **(A)** shows there were no significant differences between the music group and no sound group when accepting the fair offer of 5, and the music group have a shorter reaction time for rejecting an unfair offer than the no sound group when rejecting the moderately unfair offer 3 and 4. Panel **(B)** shows there were no significant differences when rejecting the unfair offer of 3 and 4 than accepting the fair offer of 5 in the music group, but there were significant differences when accepting the unfair offer of 3 and 4. Panel **(C)** shows the no sound group need significantly longer reaction time when rejecting or accepting the unfair offer of 3 and 4 than accepting the fair offer of 5. (*p < 0.05, **p < 0.01, ***p < 0.001).

Wilcoxon signed-rank test was conducted between reaction time of different groups, it shows that, if participants face a fair offer of 5, they quickly accept the offer, and there were no significant differences across different groups (*z* = −0.524, *p*_[FDR]_ = 0.660). Participants in the music group had shorter reaction time when rejecting the unfair offer of 3 (*z* = −2.677, *p*_[FDR]_ = 0.015) and 4 (*z* = −0.322, *p*_[FDR]_ = 0.748) than those in the no sound group (Figure 2A).

The reaction time of the music group showed that, compared with the reaction time of acceptance of fair offers, participants in the music group did not have significant different reaction time when rejecting the unfair offer of 3 (*z* = −1.754, *p*_[FDR]_ = 0.096) and 4 (*z* = −1.918, *p*_[FDR]_ = 0.076), while had significantly longer reaction time when accepting the unfair offer of 3 (*z* = −2.903, *p*_[FDR]_ = 0.011) and 4 (*z* = −2.971, *p*_[FDR]_ = 0.011) (Figure 2B).

It showed that participants in the no sound group need longer reaction time to reject (reject the unfair offer of 3: *z* = −3.416, *p*_[FDR]_ = 0.005; reject the unfair offer of 4: *z* = −2.138, *p*_[FDR]_ = 0.050) or accept all unfair offers (accept the unfair offer of 3: *z* = −3.771, *p*_[FDR]_ < 0.001; accept the unfair offer of 4: *z* = −2.417, *p*_[FDR]_ = 0.029) than to accept fair offers (Figure 2C).

### Medial frontal negativity

Independent samples *t*-tests showed no significant difference in MFN amplitude between the music and no sound groups for accepting or rejecting the moderately unfair offer. Participants in the music group did not have significantly different MFN amplitudes when accepting (*p*_[FDR]_ = 0.326) or rejecting (*p*_[FDR]_ = 0.326) the unfair offer of 3 than those in the no sound group. Participants in the music group did not have significantly different MFN amplitudes when accepting (*p*_[FDR]_ = 0.752) or rejecting (*p*_[FDR]_ = 0.752) the unfair offer of 4 than those in the no sound group.

### Brain network analyses

**FIGURE 3:**
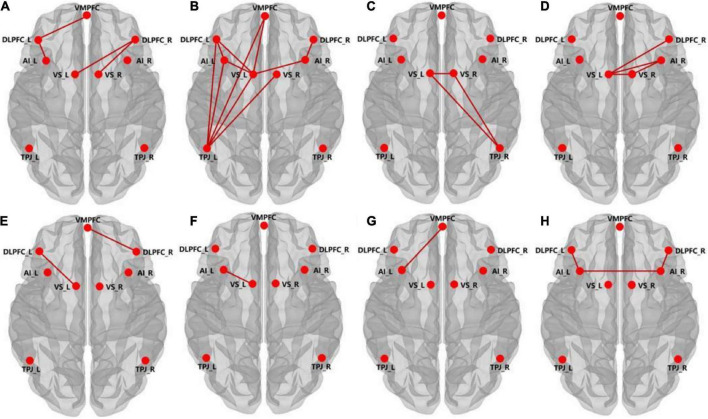
Panels **(A,B)** show the increased functional connectivities in the music group compared to the no sound group before accepting the unfair offers of 3 and 4. Panels **(C,D)** show the increased functional connectivities in the music group compared to the no sound group before rejecting the unfair offers of 3 and 4. Panels **(E,F)** show the increased functional connectivity in the music group compared to the no sound group when accepting the unfair offers of 3 and 4. Panels **(G,H)** show the increased functional connectivity in the music group compared to the no sound group when rejecting the unfair offers of 3 and 4. (Independent samples *t*-tests, all *P*s < 0.05). Abbreviations of ROIs: VMPFC, ventral medial prefrontal cortex; DLPFC_L, left dorsolateral prefrontal cortex; DLPFC_R, right dorsolateral prefrontal cortex; VS_L, left ventral striatum; VS_R, right ventral striatum; AI_L, left anterior insula; AI_R, right anterior insula; TPJ_L, left temporoparietal junction; TPJ_R, right temporoparietal junction.

Brain network analyses revealed that network connectivities between the right dorsolateral prefrontal cortex (DLPFC) and right anterior insula, bilateral ventral striatum, network connectivities between left DLPFC and VMPFC, left anterior insula, left ventral striatum, left temporoparietal junction (TPJ), network connectivities between left TPJ and left anterior insula, bilateral ventral striatum, VMPFC, as well as network connectivities between the left ventral striatum and bilateral anterior insula, VMPFC were significantly stronger in the music group than in the no sound group before accepting the unfair offers (Figures 3A,B). Network connectivities between bilateral ventral striatum, network connectivities between right TPJ and bilateral ventral striatum, network connectivities between the left ventral striatum and right anterior insula, right DLPFC, as well as network connectivities between the right ventral striatum and right anterior insula were significantly stronger in the music group than in the no sound group before rejecting the unfair offers (Figures 3C,D).

Network connectivities between right DLPFC and VMPFC, network connectivities between left DLPFC and left ventral striatum, as well as network connectivities between the left ventral striatum and left anterior insula were significantly stronger in the music group than in the no sound group when unfairly offers were accepted (Figures 3E,F). Network connectivities between bilateral anterior insula, network connectivities between left anterior insula and VMPFC, left DLPFC, network connectivities between right anterior insula and right DLPFC were significantly stronger in the music group than those in the no sound group when unfairly offers were rejected (Figures 3G,H).

In order to assess neural-behavioral correlation, based on the brain network analyses, a Pearson correlation analysis was conducted between participants’ acceptance of unfair offers and network connectivities. The results showed that the acceptance rate of unfair offers in the music group was positively correlated with the network connectivities between left TPJ and right ventral striatum before the decision-making (*p* = 0.0025, *r* = 0.7008); the acceptance rate of unfair offers in the no sound group was positively correlated with the network connectivities between right anterior insula and right DLPFC (*p* = 0.0401, *r* = 0.4406), with the network connectivities between left anterior insula and left DLPFC was negatively correlated (*p* = 0.0444, *r* = 0.4325).

## Discussion

Our study investigated the effect of absolute music on individual fairness decisions in the UG. It explored the neural activity underlying the problem by EEG to reveal the mechanism of absolute music influences fair decision-making. As we expected, absolute music can influence fair decision-making. This study chose happy music as the eliciting material, the result is inconsistent with previous studies and the hypothesis of this study. Happy music did not increase individuals’ acceptance rate of unfair offers as expected. Instead, the participants’ acceptance rate of unfair offers was significantly lower after listening to 1 min of happy music. ERP results show no significant difference in MFN amplitude between the music and no sound groups for accepting or rejecting the moderately unfair offer. Brain network analyses revealed that the networks connectivities among DLPFC, anterior insula, VMPFC and ventral striatum showed stronger activations in the music group than those in the no sound group. Next, we will discuss the implications of these findings separately.

### Absolute music can influence the outcome of fair decision-making

The behavioral results showed that the music group rejected more unfair offers compared to the control group. The behavioral and EEG results demonstrated that absolute music could influence fair decision-making at the behavioral and neuroimaging levels, respectively.

In terms of whether music can influence moral judgments, this study is consistent with previous research, although these studies have not used absolute music as an eliciting material for incidental emotions (Greitemeyer, 2009, 2011; Ziv et al., 2012; Ziv, 2016). However, the literature review suggested that absolute music as an eliciting material is the key to confirming whether music has the ethical power to influence moral judgments. This is the first study to use absolute music as eliciting material, and investigate the influences of music-induced emotions in UG and simultaneously recorded EEG data. The results facilitated our understanding of whether music can influence moral judgments.

Surprisingly, in terms of happy music reducing participants’ acceptance of unfair offers in the UG, this study is inconsistent with previous research and the hypothesis of this study. It has been noted that positive emotion induced by pictures (Liu et al., 2016), film clips (Harlé and Sanfey, 2007; Riepl et al., 2016) exert no influence on the acceptance rate of unfair offers (Harlé and Sanfey, 2007) or increased acceptance rate of unfair offers (Harlé et al., 2012; Riepl et al., 2016). The negative emotion induced by pictures (Liu et al., 2016), film clips (Harlé and Sanfey, 2007; Harlé et al., 2012; Forgas and Tan, 2013), etc., significantly reduced the acceptance rate of unfair offers in the UG.

We believe that there are two main reasons for the disagreement. First, the specificity of music allows it to influence the process of fair decision-making differently from other eliciting materials, this leads to different outcomes. Eliciting materials that have been used in studies of incidental emotions influencing fair decision-making are mainly pictures, videos, smells, etc. Studies comparing media used for the laboratory induction of emotion (film clips, still images, and music) point out that music-induced emotions are more relevant to the complex psychological processes of individuals than emotions induced by films or images, and may affect their psychological and cognitive processes in different ways (Baumgartner et al., 2006; Ellard et al., 2012; Putkinen et al., 2020). Table 1 suggests that participants in the white noise group were significantly more unhappy after listening to white noise. However, participants in both the music and the white noise groups showed a decreasing trend in ARs of unfair offers, suggesting that happy emotions induced by music and unhappy emotions induced by white noise influence fair decision-making through different mechanisms.

Second, the valence of emotions is not a critical factor in influencing decision-making. Frijda suggests that the motivational dimension of emotions, rather than the validity of emotions, is the key factor in influencing decision-making (Frijda, 1986). The valence of emotions is divided into positive emotions and negative emotions. The motivation of emotions is distinguishing emotional experience as a two-dimensional structure: approach vs. avoidance, they promote approach and withdrawal behavior, respectively (Lang et al., 1997; Harlé and Sanfey, 2010). Two emotions with similar valences may have different motivations and vice versa. Forgas also pointed out that emotional valence, motivation, arousal (Clark et al., 1984; Paulhus and Lim, 1994), and cognitive appraisal patterns (Roseman, 1984; Smith and Ellsworth, 1985) all influence decision-making. Thus, the influence of different characteristics of music on moral judgment needs to be further investigated.

### The rewarding compensatory mechanism of music reduces participants’ acceptance rates of unfair offers

Brain network analyses showed that, compared to the no sound group, brain areas closely related to decision-making, such as the DLPFC, TPJ, anterior insula, VMPFC, and ventral striatum, were significantly activated in the music group during the acceptance or rejection of unfair offers, suggesting that both cognition and emotion involved in the process of music influencing fair decision-making process, and that there was an interaction between cognition and emotion. Participants in the music group showed stronger activation of rewarding circuits, including the ventral striatum, during the decision-making process of rejecting unfair offers, before the decision especially, compared to the no sound group. Prior studies suggested that the brain mechanisms by which music-evoked pleasure is associated with the reward pathway (Koelsch, 2014). Salimpoor et al. (2013) explored the link between musical anticipation and rewarding by measuring participants’ responses to how much they would be willing to pay to hear the previous music again after first listening to unfamiliar music, and suggest that aesthetic rewards arise from the interaction between mesolimbic reward circuitry and cortical networks involved in perceptual analysis and valuation. Gold et al. (2019)’s fMRI experiments examined whether the voxel nucleus responds to reward prediction errors in music further validated the association between positive reward prediction errors and the experience of musical pleasure. Furthermore, it has been shown that the DLPFC is associated with restraining self-interest and enforcing control in fair decision-making (Knoch et al., 2006, 2008; Baumgartner et al., 2011), combined with the position of the DLPFC at the highest level of the frontal lobe hierarchical cognitive control network, responsible for top-down contextual signals to regulate stimulus-response processes (Badre and Nee, 2018). In unfair offers, participants would originally need to choose between self-interest and fairness norms (Sanfey et al., 2003; Tabibnia et al., 2008; Speitel et al., 2019), due to music-induced pleasure activates participants’ reward system, this reward activation compensates participants vicariously for the reward they receive for choosing self-interest in an unfair offer, participants no longer have to choose between self-interest and fairness norms, so participants choose to reject the unfair offer due to the negative emotions induced by the unfair offer.

This hypothesis is further supported by the difference in reaction time between groups (Figure 2). If participants were confronted with a fair offer of 5, they quickly accepted it, and no differences in reaction time were observed across experimental groups. In contrast, participants in the no sound group need significantly more reaction time to accept or reject the unfair offer than to accept the fair offer. The reason is that with unfair offers, participants need to choose between self-interest and fairness norms, resulting in increased reaction time. However, participants in the music group did not have a significantly different reaction time for rejecting an unfair offer than accepting a fair offer. Still, there was a significant difference between the reaction time for accepting an unfair offer and the reaction time for accepting a fair offer. Participants in the music group had a significantly lower reaction time for rejecting an unfair offer than those in the no sound in the unfair offer of 3.

Interestingly, although music significantly decreased participants’ ARs of unfair offers, music did not alter participants’ fairness judgments. In the UG, MFN is an ERP component that responds to levels of fairness, and unfairness offers induce greater MFN magnitudes compared to fairness offers (Hewig et al., 2011; Wu et al., 2011; Massi and Luhmann, 2015). Independent sample *t*-tests showed no significant difference in MFN amplitude between the music and no sound groups for either accepting or rejecting the moderately unfair offer. Thus, music significantly changed participants’ fair behavioral responses to unfair offers, but did not affect participants’ fairness judgments.

### Limitations and further studies

There are several limitations in our study. First, considering the millisecond-level information processing ability of the brain, EEG has more advantages in time-varying brain network analysis. However, compared to fMRI, the spatial resolution of EEG is somewhat limited. Thus, the EEG-fMRI multimodal fusion technique should be adopted in future studies to give full advantages of the complementary spatiotemporal resolution of EEG and fMRI.

Second, although we found that absolute music had an effect on fair decision-making, the music selected for our experiments was happy music which can evocate happy emotion. As happy music reduced participants’ ARs of unfair offers, it’s inconsistent with previous research on happy emotions induced by pictures or film clips. Still, it is consistent with prior research on sad emotions induced by pictures or film clips, suggesting that music-induced emotions may affect participants’ psychological and cognitive processes in different ways. It is necessary to carefully explore the influence of music (music emotion or the characteristics of music itself, such as timbre, pitch, and rhythm) on moral judgment. For example, following studies could select happy and sad music concurrently to compare the effects of positive and negative musical emotions on fair decision-making in one study.

## Conclusion

Whether music can influence moral judgment is an important topic in the aesthetics and philosophy of music. The key to resolving the debate is to investigate the brain mechanism of music influencing moral judgment with absolute music as the inducing material. In this study, we used absolute music as the eliciting material and asked participants to complete the UG after listening to the music, and explored the neural activity underlying the problem through EEG to reveal the mechanisms by which absolute music influences fair decision-making. This study concludes that absolute music can influence fair decision-making. The effect of absolute music on fair decision-making is not only the result of the co-working between incidental and integral emotions, but also the interaction of emotion and cognition. Our study provides further insights on the debate on the moral education function of music and explains the role and mechanisms of music in moral judgment. The mechanisms of music influencing fair decision-making proposed in this study also suggest that the process of emotions influencing decision-making goes beyond the single perspective of dealing with integral emotions evoked by the task, incidental emotions, or cognitive-emotional interactions in prior explanatory pathways. Future research should integrate the previous three illustrative pathways and examine how emotions influence decision-making more comprehensively.

## Data availability statement

The original contributions presented in this study are included in the article/Supplementary material, further inquiries can be directed to the corresponding authors.

## Ethics statement

The studies involving human participants were reviewed and approved by the Ethics Committee of the University of Electronic Science and Technology of China. The patients/participants provided their written informed consent to participate in this study.

## Author contributions

ZL, LY, and LH conceptualized the contribution. ZL, YS, and BH collected the data. SL, JW, and RD contributed to the formal analyses and discussion. ZL and LY wrote the first draft. JL and DY revised the manuscript critically for important intellectual content. All authors contributed to the article and approved the submitted version.

## Footnotes

1
https://www.neuro.uestc.edu.cn/name/shopwap/do/index/content/96

2
https://neuroimage.usc.edu/brainstorm/

3
http://braph.org/
