# Supplementary material for: The rewarding compensatory mechanism of music enhances the sense of fairness
Source: Front Behav Neurosci. 2022 Aug 1;16:890739. doi: 10.3389/fnbeh.2022.890739 (PMC9376466; doi:10.3389/fnbeh.2022.890739)
Supplement: Supplementary file 1 [file Data_Sheet_1.docx]

**Supplementary Material**

To illustrate that compared to white noise group, without affecting participants' fairness judgements for unfair offers, music ultimately decreases participants' acceptance rates of unfair offers due to the rewarding compensatory mechanism of happy emotions induced by music, we added the Reaction Time and MFN data of the white noise group vs. music group and the white noise group vs. no sound group as supplementary materials.

| Table S1 Difference in reaction time between the music and white noise groups | | | | |
| --- | --- | --- | --- | --- |
|  | music | white noise | Z | P_[FDR]_ |
| accept 5 | 660.855±105.031 | 747.614±135.560 | - 2.482 | 0.038 |
| reject 3 | 700.958±137.955 | 938.001±390.539 | - 2.907 | 0.024 |
| reject 4 | 825.319±303.226 | 1287.758±640.290 | - 2.346 | 0.038 |

Table S1 shows the music group has a shorter reaction time for accepting a fair offer than the white noise group， and the music group has a shorter reaction time for rejecting an unfair offer than the white noise group when rejecting the moderately unfair offer 3 and 4.

| Table S2 Difference in reaction time between the no sound group and white noise groups | | | | |
| --- | --- | --- | --- | --- |
|  | no sound | white noise | Z | P_[FDR]_ |
| accept 5 | 676.530±92.439 | 747.614±135.560 | - 2.072 | 0.057 |
| reject 3 | 904.301±300.984 | 938.001±390.539 | - 0.089 | 0.929 |
| reject 4 | 1045.922±795.734 | 1287.758±640.290 | - 1.775 | 0.092 |

Table S2 shows the no sound group has a shorter reaction time for accepting a fair offer than the white noise group， and there were no significant differences between the no sound group and the white noise group when rejecting the moderately unfair offers 3 and 4.

| Table S3 Difference in MFN between the music and white noise groups | | | | |
| --- | --- | --- | --- | --- |
|  | music | white noise | F | P_[FDR]_ |
| reject 3 | 5.588±2.249 | 5.433±1.631 | 3.464 | 0.396 |
| reject 4 | 6.208±1.600 | 6.885±2.090 | 0.983 | 0.503 |
| accept 4 | 5.510±3.036 | 5.591±1.803 | 2.425 | 0.396 |

| Table S4 Difference in MFN between the no sound group and white noise groups | | | | |
| --- | --- | --- | --- | --- |
|  | no sound | white noise | F | P_[FDR]_ |
| reject 3 | 4.477±1.737 | 5.433±1.631 | 0.034 | 0.956 |
| reject 4 | 5.723±2.874 | 6.885±2.090 | 0.003 | 0.956 |
| accept 4 | 5.862±2.406 | 5.591±1.803 | 1.345 | 0.503 |

Table S3 and Table S4 show no difference in MFN amplitude between the music and white noise groups or the no sound and white noise groups in any conditions. It indicates that music did not affect participants' fairness judgments.
